# The COVID-19 pandemic’s impact on mental health care use among Norwegian students in higher education: a nation-wide register-based pre-post cohort study

**DOI:** 10.1186/s12913-022-08816-3

**Published:** 2022-12-10

**Authors:** Mari Grøsland, Vilde Bergstad Larsen, Anne Reneflot, Rannveig Kaldager Hart

**Affiliations:** 1 Division for Health Services, Cluster for Health Services Research, Norwegian Institute of Public Health, Postboks 222, Skøyen, 0213 Oslo, Norway; 2 Department for Mental Health and Suicide, Division of Mental and Physical Health, Norwegian Institute of Public Health, Oslo, Norway; 3 Department for Health and Inequality and Centre for Evaluation of Public Health Measures, Division of Mental and Physical Health, Norwegian Institute of Public Health, Oslo, Norway

**Keywords:** Students, Mental health, Primary health care, Prescription drugs, COVID-19

## Abstract

**Background:**

The COVID-19 pandemic, and its associated social distancing measures, gave profound changes to the everyday and academic life of students in higher education. The current study is the first to use nation-wide data to evaluate the long-term effect of the pandemic and its countermeasures on university students’ mental health care use.

**Methods:**

Using nation-wide individual-level data, we studied mental health consultations in primary care (data available from January 2017 to February 2022) and dispensed prescription drugs used to treat anxiety, depression, and sleep disturbances (data available from October 2018 to February 2021) for first-year undergraduate university students. We compared changes over time in mental health care use in a pandemic cohort (12,501 first-year students enrolled in 2019) to the same change in a pre-pandemic cohort (25,990 first-year students enrolled in 2017 and 2018). Event study and difference-in-difference models allowed us to separate the impact of the pandemic, experienced by the pandemic cohort only, from secular and seasonal changes experienced by all cohorts.

**Results:**

The percentage of students with a mental health consultation temporarily decreased during the first period of strict social distancing measures in March 2020. At the end of the second round with strict measures in April 2021, the level of mental health consultations increased by 73% (95% CI 40–106.3). There was also a 42% (95% CI 5.7–79.5) increase in mental health consultations in November 2021. No similar increases were observed for dispensed prescription drugs between March 2020 and February 2021.

**Conclusions:**

The COVID-19 pandemic was associated with increases in mental health consultations in primary care among students, especially during/after longer periods of strict social distancing measures. The benefits of social distancing measures in future pandemic preparedness should be weighed against the cost of potentially worsening mental health in vulnerable groups.

**Supplementary Information:**

The online version contains supplementary material available at 10.1186/s12913-022-08816-3.

## Introduction

**Fig. 1:**
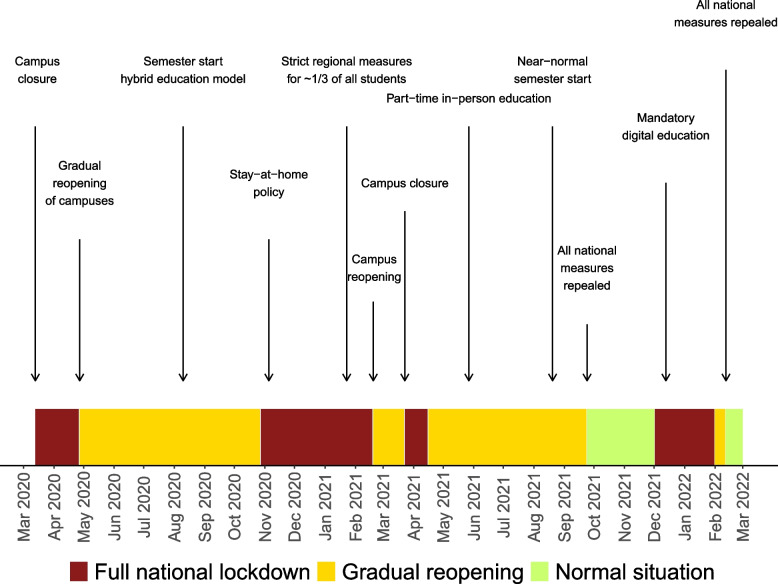
Timeline of COVID-19 restrictions nationally implemented in Norway, affecting university students. The timeline illustrates the infection control measures implemented nationally by the Norwegian government to limit the spread of SARS-CoV-2 from the first national measures in place from March 13^th^, 2020, to all national measures were repealed on February 12^th^, 2022. The measures are categorized by level of stringency. The category ‘Full national lockdown’ refers to the strictest sets of injunctions and recommendations that included e.g., campus closure (including libraries); stay-at-home orders; limits on the number of visitors; closure of non-essential shops, bars, restaurants, and gyms; limitations on number of attendees at public events; hygiene and social distancing measures (e.g., 1- or 2-m distance in public and private spaces, face mask requirements); and testing, quarantine, and isolation rules. The category ‘Gradual reopening’ refers to periods after full lockdown, where some social distancing measures were gradually lifted, while e.g., hygiene, public distance, quarantine, and isolation rules remained. Typically, this involved a hybrid education model with part-time digital and part-time physical education for university students. The category ‘Normal situation’ refers to periods where all invasive national measures (except e.g., testing, quarantine, and isolation rules) were nationally repealed [2]

In early March 2020, governments across the world implemented strict social distancing measures and stay-at-home orders to limit the spread of SARS-CoV-2, the virus that caused the global COVID-19 pandemic. As a result, higher education facilities and campuses were commonly closed, disrupting the regular educational activities for millions of university students worldwide [1]. After the first wave of the pandemic, the pace of reopening varied vastly across the globe. In Norway, a gradual reopening of society and university campuses was initiated in late April 2020 (see timeline in Fig. 1). With increasing COVID-19 infection rates in the fall of 2020, restrictions were reintroduced, lasting for a total of almost two years with varying levels of stringency over time [2].

Restrictions and stay-at-home policies may be particularly harmful for young university students as they are already going through major life changes such as moving away from home, establishing new social networks, and pursuing higher education [3–6]. Studies mainly based on convenience samples and cross sectional data suggest that the initial shock of the pandemic and the associated restrictions in the spring of 2020 had an impact on students’ mental health such as increased levels of stress, anxiety, depression, insomnia and general well-being [4, 7–13]. In contrast, results for the general population are more mixed, and worsened mental health (if any) tend to emerge later in the pandemic [14–17]. Furthermore, there has been steep increase in mental health symptoms among (particularly female) students prior to the pandemic, sometimes assessed to be sharper than among their non-enrolled peers [18]. This suggests that students may be particularly vulnerable to deteriorating mental health during a pandemic. Any deterioration of mental health among students may also have severe and potentially long-lasting consequences: Mental health symptoms and disorders that emerge at these ages have higher risk of relapse and recurrence in later life [19–21]. Beyond the reduction in quality of life, a range of mental illnesses are found to negatively affect students’ academic performance and increases the risk of dropping out [22], in turn limiting occupational and financial opportunities. More knowledge on the impact of the pandemic on student’s mental health is therefore important input to pandemic preparedness for the future. Moreover, to provide adequate support systems and mend adverse effects, policy makers need to know how the pandemic has changed the demand of mental health care services among students in higher education.

To our knowledge, the current study is the first to use representative, nation-wide register data to describe and assess the relationship between the pandemic with longer periods of strict social distancing measures and first-year students’ health care use related to mental health from 2017 to February 2022. Mental health symptoms and disorders often emerge in late adolescence and early adulthood [19]. To separate the effect of the pandemic from a general trend of increasing mental health issues and health care use over age, we compared the change in mental health outcomes of students who experienced the pandemic to the change in a prior cohort of students who did not. As the outcomes we study are more prevalent in females than males [18], we also assessed whether changes during the pandemic varied by sex.

## Data and methods

We utilized nation-wide individual-level register data from the Norwegian Emergency Preparedness Register, Beredt C19, originating from the following registers: the Norwegian Population Register (demographic characteristics, including date of birth and sex); Statistics Norway (student status); the Norway Control and Payment of Health Reimbursement Database (primary care consultations and e-consultations at general practitioner or emergency ward, available from January 2017 to February 2022) and the Norwegian Prescription Database (all prescription drugs dispensed in Norwegian pharmacies to non-institutionalized individuals, available from October 2018 to August 2021). The data sources were linked using a deidentified version of the personal identification number received upon birth or immigration to Norway.

### Study population

**Fig. 2:**
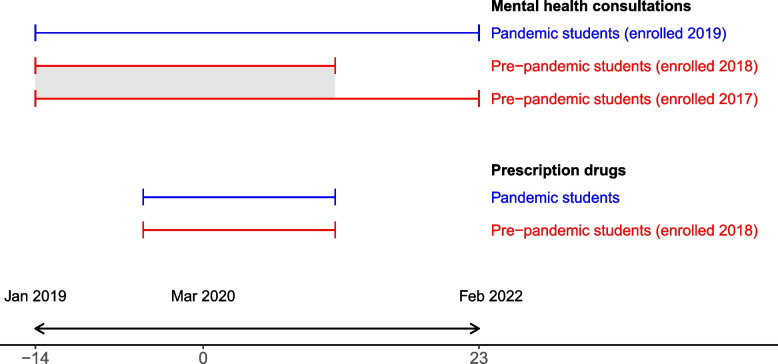
Observation period for the pandemic cohort and pre-pandemic cohort across outcomes. We compared the percentage with a mental health consultation/prescription between the pandemic and pre-pandemic students each relative month, to ensure comparison between same-aged students. The start of the pandemic, March 2020, is set as relative month 0. Relative months -14 to -1 constituted the pre-treatment period and relative months 0 to 23 constituted the treatment period. In relative months that overlapped between the two cohorts that constituted the pre-pandemic cohort (shaded area), we calculated the average outcome value for descriptive statistics. Due to data availability, prescription drugs could only be observed between relative months -5 and 11

Our study population for outcomes in primary care (consultations related to mental health) consisted of all first-year undergraduate students who were enrolled in higher education in the fall of 2017, 2018, and 2019. First-year students were defined as students who turned 19 in the year of enrollment and who were registered as graduating from high school in the spring of the same year. Only students enrolled in 2019 were observed in the pandemic, thus forming our pandemic cohort. The pandemic cohort was observed for a total of 38 months, starting in January 2019, through February 2022, when national COVID-19 measures were repealed. The pre-pandemic cohort consisted of students enrolled in 2017 and 2018 who were observed from January in the year of enrollment (January 2017 and 2018, respectively), until February 2020, the last month before COVID-19 infection control measures were first implemented. Hence, the pre-pandemic cohort was observed 24 and 12 months before the pandemic cohort (Fig. 2). Students in both cohorts who died or emigrated were observed until the month of death or emigration (approximately 0.05% of all person months were excluded). Due to our availability of prescription of drugs data from the Norwegian Prescription Database, the study population for prescribed drugs was limited to first-year undergraduate students enrolled in higher education the fall of 2018 and 2019. The pandemic cohort (students enrolled in 2019) was observed from October 2019 through February 2021, while the pre-pandemic cohort (students enrolled in 2018) was observed from October 2018 through February 2020 (Fig. 2).

### Outcomes

Our primary outcome of interest was primary care consultations coded with a mental health symptom or diagnosis. To capture specific symptoms of mental health problems commonly reported among students during the pandemic, we also measured consultations separately for anxiety, depression, and sleep disturbances (see Table 1 for the specific ICPC-2 codes used to define outcomes). Anxiety and depression have high levels of comorbidity, and these were therefore studied jointly [19, 23, 24]. In addition, we measured dispensed prescription drugs typically used to treat anxiety (anxiolytics), depression (antidepressants), and sleep disturbances (sedatives). While consultations can be related to symptoms both above and below the clinical threshold, prescriptions are largely restricted to more severe outcomes (see Table A1 for specific ATC codes used). In Norway, anxiolytics, antidepressants, and sedatives are prescribed by the general practitioner and typically requires at least one primary care consultation. We measured each outcome separately and constructed monthly measures that were set to one if the student had at least one consultation or dispensed prescription drug the given month, and zero otherwise.

**Table 1:** Descriptive statistics on group characteristics for pre-pandemic and pandemic students

	**Pandemic cohort**		**Pre-pandemic cohort**		
**Sample characteristics**					
Persons, N	12 501		25 990		
Age, mean (SD)	19 (0)		19 (0)		
**Sex, N (%)**					
Females	7 878 (63.0)		16 241 (62.5)		
Males	4 623 (37.0)		9 749 (37.5)		
**Birth country, N (%)**					
Norway	11 192 (89.5)		23 587 (90.8)		
Abroad	1 309 (10.5)		2 403 (9.2)		
**Mental health outcomes, monthly %**	**Jan 2019-Feb 2020**	**Mar 2020-Feb 2022**	**Jan 2019-Feb 2020**^a^	**Mar 2020-Feb 2022**^b^	
**Primary care consultations**					**ICPC-2 codes**
Any mental symptom or disorder	1.15	1.25	1.17	1.38	All chapter P codes
Anxiety, depression	0.64	0.72	0.66	0.86	P01, P02, P03, P74, P76, P79, P82
Sleep disturbance	0.17	0.13	0.17	0.14	P06

Mental health outcomes show the monthly percentage of pandemic and pre-pandemic students with a mental health consultation. See table A1 for the corresponding table for dispensed prescription drug (anxiolytic, antidepressant, or sedative). In primary care reimbursement codes 2ad, 2ak, 2ae is used to identify consultations at general practitioner or emergency ward

^a,b^January 2019-February 2022 refers to the measurement time (calendar month) for the pandemic students, i.e., measurements for the pre-pandemic students were made 12 and 24 months earlier. ^a^January 2019-February 2020 corresponds to relative months -14 to -1 (pre-treatment period). ^b^March 2020-February 2022 corresponds to relative months 1 to 23 (post-treatment period)

### Intervention/treatment

Our intervention of interest was the COVID-19 pandemic, defined as starting in March 2020 at the start of the first lockdown. Only the pandemic cohort was observed during the pandemic.

We separated the effect of the pandemic from other temporal trends by comparing the change in the monthly percentage of students in the pandemic cohort with mental health outcomes to the change in the percentage among same-aged students in the pre-pandemic cohort. We defined a duration variable (*t*) set to zero in the first month of the pandemic, i.e., March 2020 for the pandemic cohort enrolled in fall 2019 (Fig. 2). For the pre-pandemic cohort, *t* was set to zero in March 2019 for those who enrolled in fall 2018 and March 2018 for those who enrolled in fall 2017. In part of our analysis, when focusing on seasons instead of months, the duration variable for months is collapsed into seasons, counting number of seasons (3-month groups) from t0.

### Statistical method

First, we calculated and presented descriptive statistics on sample characteristics and outcomes for the pandemic cohort and the pre-pandemic cohort before and after the intervention. For all outcomes, we plotted the monthly percentages of students with at least one consultation or dispensed drug prescription each month varied by the duration variable. We also plotted the level of COVID-19 restriction measures to assess any temporal associations.

Second, we used an event study model to isolate the effect of the pandemic on students’ mental health care use, by effectively netting out any seasonal changes as well as changes related to the study course. Month by month, we evaluated whether changes in consultations or dispensed drugs related to mental health for the pandemic cohort differed from changes in consultations or dispensed drugs for the pre-pandemic cohort. In this model, the month prior to the COVID-19 pandemic (February 2020) was set as reference. We used data from the 14 months before the pandemic (January 2019 to February 2020 for the pandemic cohort) to assess whether the trends were comparable in the pandemic and pre-pandemic cohorts before the onset of the pandemic (i.e., to assess the parallel-trends assumption). To quantify the magnitude of the effect of the pandemic, we also estimated a difference-in-difference regression model with separate estimates for each season from March 2020 to February 2022 (March–May (spring); June–August (summer); September–November (fall); and December-February (winter)) and compared it to the 14 months before the pandemic. This allowed us to compare the percentage of pandemic students with at least one mental health consultation or drug prescription to the same monthly percentage among same-aged pre-pandemic students. In addition to the main analysis of all pandemic and pre-pandemic students, we conducted a subsample analysis with the student cohorts stratified by sex.

In all regression models (event study and difference-in-difference), we controlled for sex (apart from in the subgroup analysis). Dummy variables for year of enrollment controls out differences between the students enrolled in 2017, 2018 and 2019, including differences between the pandemic and pre-pandemic cohort. We also include a set of dummy variables for duration in month (duration in season for our difference-in-difference regressions), controlling out trends over time in consultation frequency shared between the three cohorts. Our coefficient of interest are the interactions between duration in month (duration in season for our difference-in-difference regressions), and the dummy for pandemic-cohort. These estimates show how trend over time in the pandemic cohort deviate from the trend over time in the pre-pandemic cohort. The regression estimates were presented for each month/season as a change in percentage points. The corresponding 95% confidence intervals (CIs) were clustered on the individual level to account for within-person correlation across time. We also calculated the relative change in percent by dividing the absolute estimate by the monthly average of the health outcome for the pandemic students in the period prior to the pandemic (January 2019-February 2020 for consultations, October 2019-February 2020 for prescribed drugs), multiplied by 100.

## Results

Among 38,491 first-year students enrolled between 2017 and 2019, we studied 12,501 pandemic students (enrolled in 2019) and 25,990 pre-pandemic students (enrolled in 2017 and 2018) (Table 1). The pandemic and pre-pandemic students shared similar characteristics, both consisting of a large majority of Norwegian-born females. On average, there were minor differences in the monthly percentage with a mental health consultation/prescription between the two cohorts. The monthly percentage with a mental health consultation or prescription increased slightly from the pre-treatment period to the post-treatment period in both cohorts and for most outcomes, i.e., from before to after the onset of the pandemic for the pandemic students (Table 1). Table A1 shows the corresponding table for the dispensed prescription drugs sample.

### Mental health consultations and dispensed prescription drugs over time

**Fig. 3:**
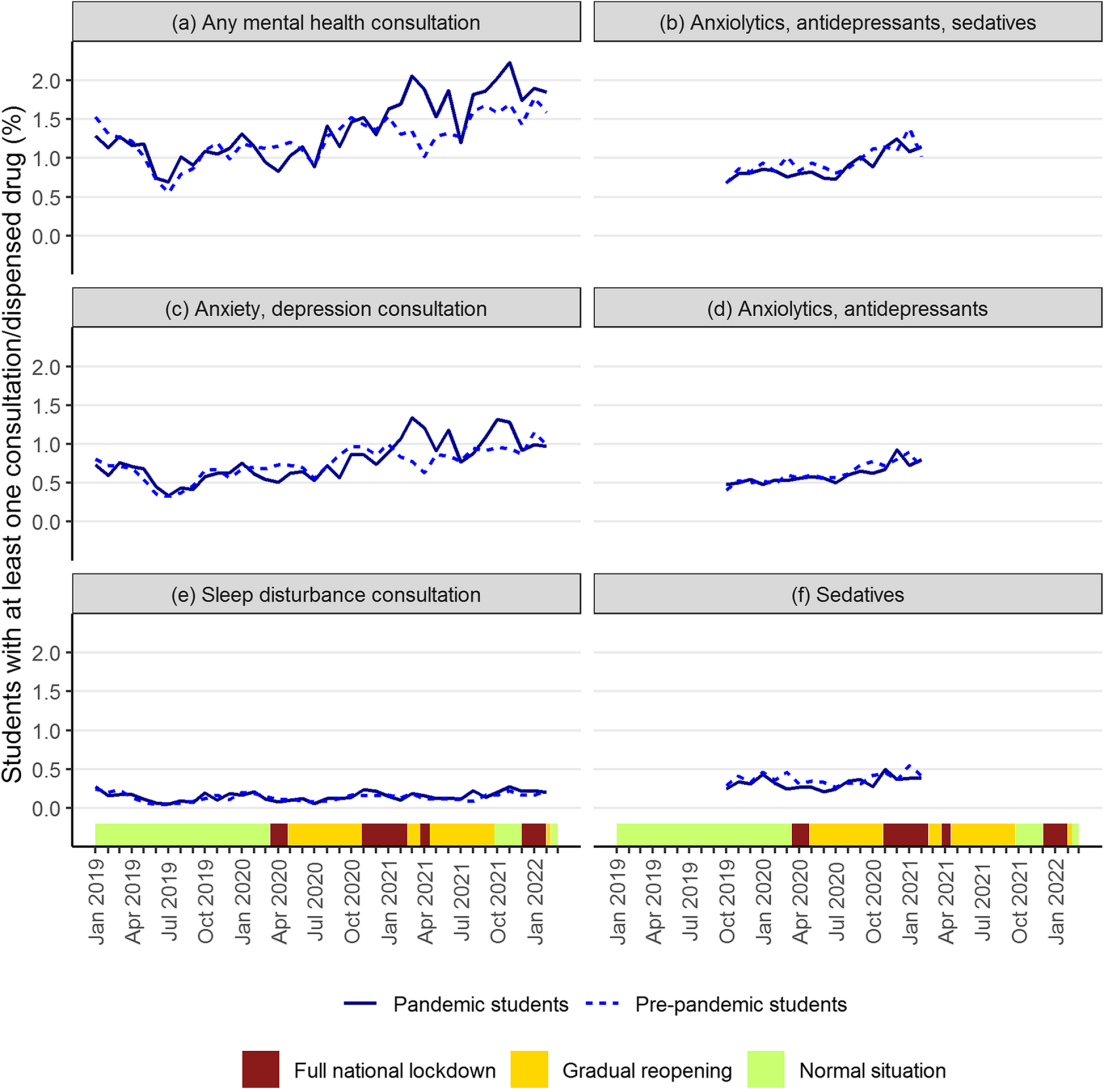
Crude monthly percentages of mental health consultations and dispensed drugs among students. All outcomes were measured and calculated separately for the pandemic students (solid lines) and pre-pandemic students (dashed lines). The monthly percentages refer to students with at least one mental health consultation or dispensed prescription drug the given month. The x-axis refers to the calendar month where measurements were made for the pandemic students. The pre-pandemic students were measured 12 and 24 months earlier. The timeline of infection control measures, categorized by three levels of stringency, is illustrated on the x-axis (analogous to Fig. 1)

The percent of students who sought mental health care varied over time, from around 0.5 to 2% (Fig. 3). For both cohorts, the monthly share of students with a mental health consultation increased throughout the study course, from the year of enrollment to the second and third years of study (Fig. 3). There was also considerable seasonal variation in consultations, with temporary decreases in the summer months, while less seasonal variation was observed for dispensed prescription drugs.

From September 2019 to March 2020, trends in mental health consultations and dispensed prescription drugs were comparable between the pandemic student cohort (solid lines) and the pre-pandemic cohort who were observed 12 and 24 months earlier (dashed lines) (Fig. 3a, b). For the pandemic students, there was a clear dip in mental health consultations, most evident for anxiety/depression consultations, in the start of the pandemic continuing into the first national lockdown, compared to the pre-pandemic students (Fig. 3a, c). There was no similar significant decrease in the crude percentage who dispensed anxiolytics, antidepressants, and sedatives (when studied jointly) during the first lockdown (Fig. 3b). The percentage who sought mental health care increased to the level of the pre-pandemic students during the first gradual reopening of society and into the second period of strict social distancing (May to December 2020) (Fig. 3a). In January 2021, the monthly percentage of pandemic students with a mental health consultation surpassed the percentage of pre-pandemic students and stabilized on a higher level thereafter, with a crude difference of 0.86 percentage points in April 2021 (Fig. 3a). This pattern was found only for anxiety and depression consultations, and not for sleep disturbance consultations (Fig. 3c, e). Due to data availability, trends in drug prescriptions could not be observed at the end of the second lockdown (after February 2021).

### The impact of the pandemic on mental health consultations and dispensed prescription drugs

To formally test whether mental health outcomes differed between the pandemic and pre-pandemic students after the onset of the COVID-19 pandemic, we estimated event study models, netting out seasonality and secular change over time.

**Fig. 4:**
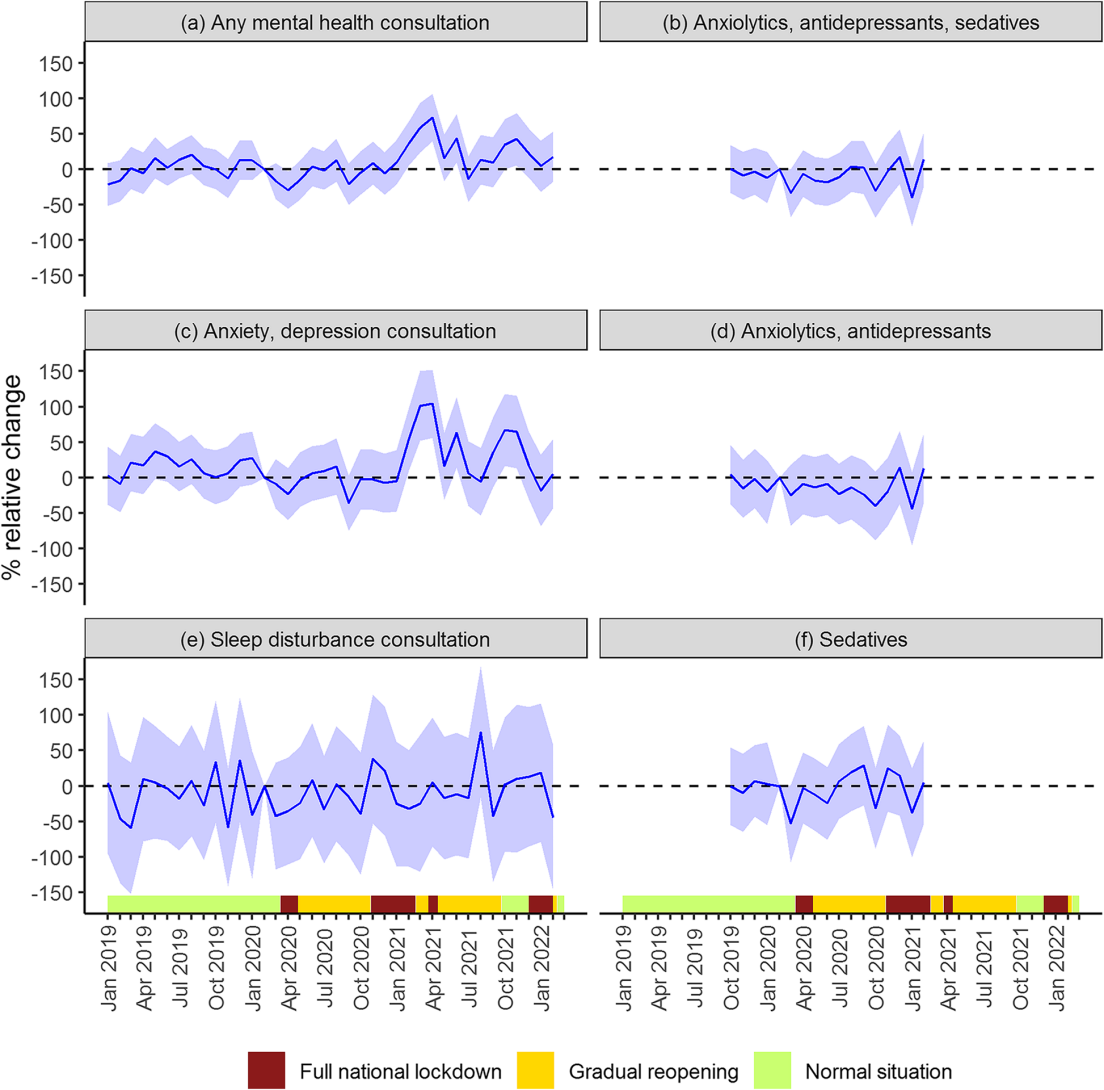
The impact of the pandemic on mental health care use among students. Results from event study models estimated separately for each outcome. Solid lines show monthly coefficients with corresponding 95% confidence interval (shaded). Coefficients refer to the monthly percentage change in mental health care use among pandemic students, relative to pre-pandemic students. See Appendix figure A1 for the corresponding figure for absolute change (measures in percentage points). For more information on model specification see table note in table A2. The timeline of infection control measures, categorized by three levels of stringency, is illustrated on the x-axis (analogous to Fig. 1)

The results from the event study model confirmed similar trends in mental health consultations and dispensed prescription drugs between the pandemic and the pre-pandemic students before the pandemic, as the 95% CI included zero in all months (Fig. 4). In April 2020, during the first national lockdown, mental health consultations temporarily decreased with 29% among pandemic students, relative to the pre-pandemic students measured 12 and 24 months earlier, and thereafter leveled off until January 2021 (Fig. 4a, Table A2). In February through April 2021, there was a significant monthly increase in consultations of between 37 and 73%. A similar increase was also found in October and November 2021 (Fig. 4a, Table A2). These increases were driven by changes in consultations for anxiety and depression symptoms and diagnoses (Fig. 4c, e). For example, monthly anxiety and depression consultations increased by 102% and 104% in March and April 2021, respectively. There were no significant changes in monthly sleep disturbance consultations throughout the pandemic. Trends in dispensed anxiolytics, antidepressants, and sedatives did not significantly differ between the pandemic and pre-pandemic students after March 2020 until February 2021 (Fig. 4b, d, f).

For a more succinct quantitative summary of the results, we present results for a difference-in-differences model, where effects are allowed to vary by season. The estimates show a significant reduction in monthly mental health consultations (any symptom or disorder) in the spring of 2020 of 0.24 percentage points, corresponding to a 22% relative decrease (Table 2). There were no statistically significant differences in mental health consultations until spring 2021, when consultations increased by 47%, driven by a 59% increase in anxiety and depression consultations. As in the event study model, mental health consultations increased by 27% in fall 2021 (Table 2). Overall, there was a greater relative increase in consultations coded with anxiety or depression symptoms or diagnoses than for all-cause mental health consultations (Table 2). The monthly percentage of students who dispensed an anxiolytic, antidepressant or sedative was unchanged from the start of the pandemic throughout winter 2021 (Table 2).

**Table 2:** The impact of the pandemic on mental health outcomes among students, by season

	**Pre %**	**Spring 2020**		**Summer 2020**		**Fall 2020**		**Winter 2020/21**		**Spring 2021**		**Summer 2021**		**Fall 2021**		**Winter 2021/22**	
**Mental health outcome**		**β (SE)**	**Rel. %**	**β (SE)**	**Rel. %**	**β (SE)**	**Rel. %**	**β (SE)**	**Rel. %**	**β (SE)**	**Rel. %**	**β (SE)**	**Rel. %**	**β (SE)**	**Rel. %**	**β (SE)**	**Rel. %**
**Any mental health consultation**	**1.08**	-0.24*** **(0.08)**	**-22**	**0.03** **(0.08)**	**3**	**-0.08** **(0.09)**	**-8**	**0.12** **(0.09)**	**11**	0.51*** **(0.11)**	**47**	**0.14** **(0.11)**	**13**	0.29** **(0.12)**	**27**	**0.14** **(0.11)**	**13**
Anxiety, depression consultation	0.59	-0.16*** (0.06)	-27	-0.03 (0.06)	-5	-0.17** (0.07)	-29	0 (0.07)	-1-0,73	0.35*** (0.09)	59	0.04 (0.08)	6	0.25*** (0.09)	41	-0.08 (0.09)	-13
Sleep disturbance consultation	0.15	-0.03 (0.02)	-23	0.01 (0.02)	4	0.01 (0.03)	6	0 (0.03)	-1	0 (0.03)	-1	0.04 (0.03)	27	0 (0.04)	1	0.01 (0.04)	7
**Anxiolytics, antidepressants, sedatives**	**0.8**	**-0.11** **(0.07)**	**-14**	**-0.03** **(0.08)**	**-4**	**-0.04** **(0.09)**	**-5**	**0.02** **(0.1)**	**2**								
Anxiolytics, antidepressants	0.51	-0.05 (0.06)	-9	-0.04 (0.06)	-9	-0.11 (0.08)	-21	0.01 (0.08)	1								
Sedatives	0.33	-0.07 (0.05)	-23	0 (0.05)	0	0.02 (0.05)	8	-0.02 (0.06)	-6								

The first column shows the average monthly percent of pandemic students with at least one mental health outcome before the pandemic (i.e., before March 2020), calculated separately for each outcome. Difference-in-difference estimates (β) quantify the change in mental health outcomes (measured as change in percentage points), controlling for age, year of enrollment and a dummy variable for the duration variable in season-year. Standard errors (SEs) are clustered on individuals. In addition to the presentation of results in absolute terms, relative differences in percent (Rel. %) are also presented, calculated by dividing the absolute estimate (and corresponding standard error) for each of the post-periods by the monthly average health outcome for the pandemic students in the period prior to the pandemic (January 2019-February 2020 for consultations and October 2019-February 2020 for dispensed prescription drugs, Pre %). Stars indicate confidence levels (*p ≤ 0.1; **p ≤ 0.05; ***p ≤ 0.01)

### Subgroup analysis: effects by sex

**Fig. 5:**
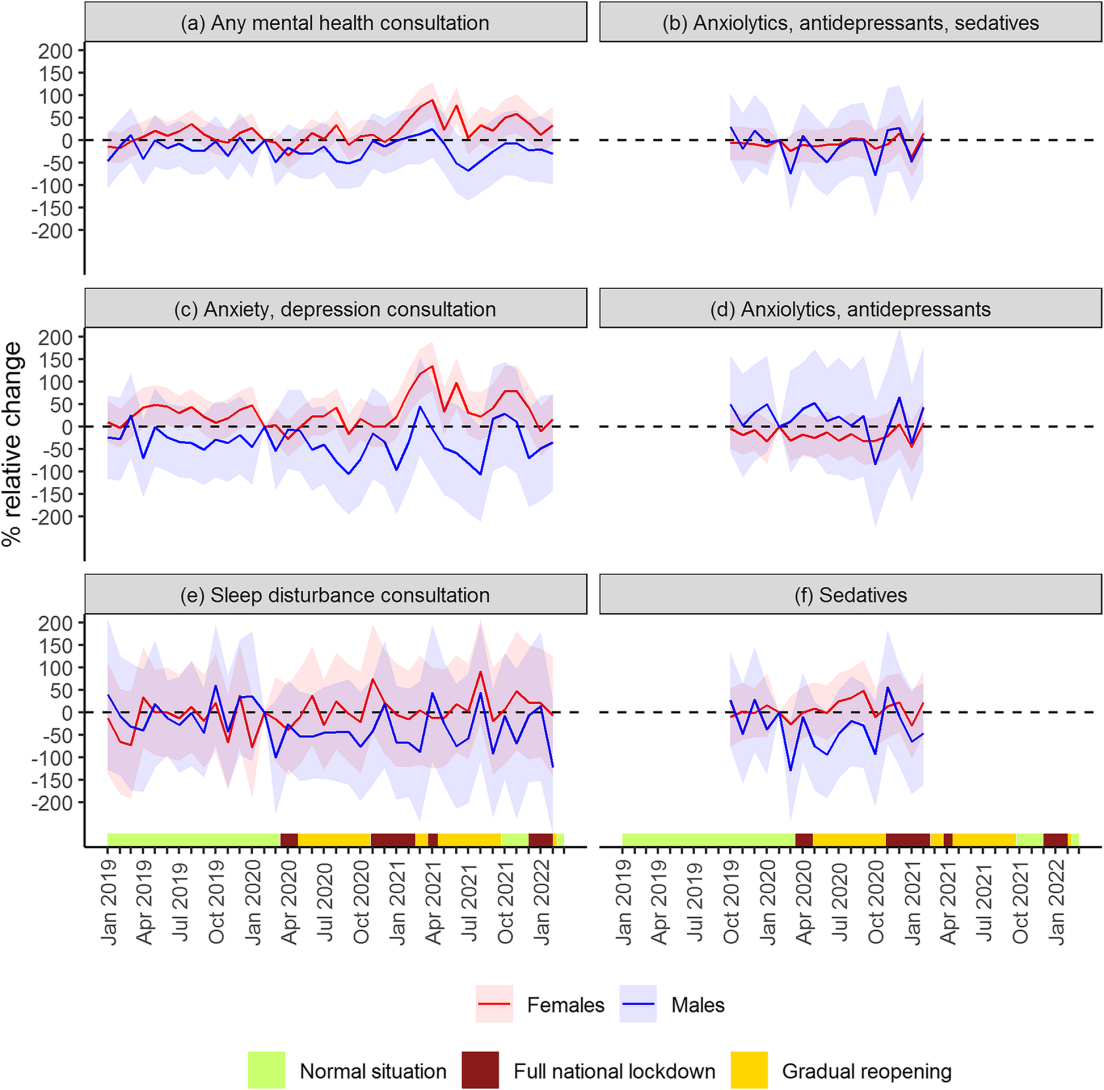
Results from event study models, stratified by sex. Results from event study models estimated separately for each outcome, sample stratified by sex. Solid lines show monthly coefficients with corresponding 95% confidence interval (shaded). Coefficients refer to the monthly percentage change in mental health care use among pandemic students, relative to pre-pandemic students, controlling for year of enrollment and a dummy for the duration variable in month. The timeline of infection control measures, categorized by three levels of stringency, is illustrated on the x-axis (analogous to Fig. 1)

The results from the event study model estimated separately for female and male students showed that the changes in mental health outcomes were driven by female students (Fig. 5). The increase in mental health consultations, specifically anxiety and depression consultations, at the end of the second period of strict social distancing measures was only observed for female students (Fig. 5a, c). No significant changes were observed for dispensed anxiolytics, antidepressants, or sedatives for either sex (Fig. 5b, d, f). As in the main model, no changes were observed for sleep disturbance consultations throughout the pandemic.

## Discussion

In the current study of 12,501 students in Norwegian higher education who were exposed to the pandemic in their first year of study, we have shown a statistically significant increase in mental health consultations in primary care in the spring and fall of 2021, compared to the increase in a similar period for 25,909 pre-pandemic first year students. The increases in mental health consultations were driven by increases in anxiety and depression consultations and were higher among female students. No significant changes were observed for dispensed anxiolytics, antidepressants, or sedatives, measured until February 2021. Our results can be driven both by an increasing number of students being in contact with the health care system, and by more frequent contact among those who were already in contact prior to the pandemic.

To our knowledge, this is the first study to use nation-wide data to examine the long-term impact of the COVID-19 pandemic on mental health care use in primary care among first-year students in higher education. Comparable studies have typically focused on children or the general population [25, 26] while studies on mental health issues due to the pandemic that focus on students in higher education are limited to survey and interview studies based on convenience sampling and self-reporting of symptoms and are mainly cross-sectional studies conducted in the first phase of the pandemic [4, 7–11].

In line with other studies on use of health services during the pandemic, we show that the level of mental health consultations was reduced relative to previous years in the spring of 2020 [27–29]. This finding is, however, in contrast to survey studies that reported higher prevalence than before of psychological distress, including symptoms such as stress, anxiety, sleep pattern disruption, and depressive thoughts, at the beginning of the pandemic [7]. A diagnostic population-based study for Norway for the same period, on the other hand, found no increase during lock-down [30], suggesting that the higher prevalence in this period is restricted to symptoms and/or differs between the general population and convenience samples. The relative reduction in consultations may also be explained by a higher threshold to seek health care at the beginning of the pandemic, due to e.g., fear of infection, limited health care capacity, or fear of overloading health care capacity. This may lead to increased shares of untreated symptoms and aggravated symptom severity ultimately leading more students to seek medical help in later phases.

Increasing demand for mental health care among university students was closely related to longer periods of strict infection control measures. While this is not evidence of a causal relationship, the temporal proximity to the relative increase in mental health care use is striking. Another potential explanation of the co-occurrence is increases in contagion in the same time periods. However, given that mortality did not increase during the pandemic in Norway [31], we consider that grief, or fear of the loss of a loved one are less likely mechanisms in this context. The largest increase in mental health consultations was found in April 2021, at the end of a long period of strict social distancing measures. This finding may indicate substantial pandemic fatigue and is consistent with other studies reporting that the psychological burden of the pandemic was higher during the second lockdown than during the first in March/April 2020 [32], implying that both the level of strictness and duration of restrictions, may have negatively impacted students’ mental health. As for the absence of effects on prescription drugs, we note that these outcomes are observed only in the period before effects on consultations emerge. Furthermore, it is well known that the road from psychological symptom onset to professional treatment may be long [33, 34], so that if anything, effects on prescriptions will emerge with a lag.

The increase in mental health consultations in primary care is substantially larger than what is found for slightly younger adolescents in Norway (aged 16–19) in a similar design [27]. However, in this study the modest effects among high school students could in part be linked to that the requirement of GP certification for sickness absence was relaxed in this age group during the pandemic. It is also possible that the students new to higher education and just starting their lives as young adults were particularly vulnerable to detrimental mental health effects of social distancing. This would be in line with a study showing more stressors and higher levels of mental health issues among university students than among non-student peers during the pandemic [5]. Furthermore, university students were hit harder by social distancing than their slightly younger high school peers: High school students adolescents tend to live with their parents, which may have contributed to less loneliness and more structured everyday life, and remote learning was used more extensively for university students than the slightly younger high school students. Additionally, many students in Norway depend economically on part-time work in sectors that were most frequently and severely affected by temporary layoffs during the pandemic, leading to substantial economic uncertainties and worry. Survey results from prior to the pandemic show associations between financial vulnerability, low academic self-efficacy, delayed study progress, loneliness, and symptoms of severe mental health problems [35].

Strengths of the current study include of nation-wide, longitudinal register-based data in a setting with universal access to both higher education and health care. Low or zero cost of consultations in Norway means that changes in mental health can be quick to manifest as changes in the demand for health care, as lack of economic resources do not hinder students in seeking help. Additionally, by comparing mental health care use for students who were exposed to the pandemic to same-aged non-pandemic students we isolate the impact of the pandemic from secular trends in health service use by both age, duration of study and period in a novel way. Furthermore, the inclusion of both symptom and diagnosis codes related to mental health reduces the potential bias of misclassifications and inconsistencies in coding practices between general practitioners.

There are also important limitations to this study. First, we use change in primary care consultations as a proxy for change in mental health. About 70% of those with an anxiety or depression diagnosis have a primary care mental health consultation over a three year period in Norway [36]. Thus, our measure can be expected to broadly, albeit with some undercount [37, 38], capture changes in underlying mental health. It is of course also possible that discontinuous changes in consultation frequency during the pandemic are driven by causes other than changes in underlying health. While fear of contagion upon personal contact could increase the threshold for seeking help, the access to e-consultations increased rapidly after the onset of the pandemic, and these consultations are included in our data. As such, a higher threshold for seeking help is unlikely to bias our estimates downwards. While e-consultations could in principle lower the threshold for seeking health care, we do not have any indication that this should systematically co-occur with severity of social distancing measures. Similarly, as a range of COVID-19 restrictions were implemented at once in Norway, we cannot pin down empirically exactly what component of the restrictions that impacted students’ mental health. To interpret the temporal co-occurrence, we must rely on previous studies and in-depth knowledge on the Norwegian context. Second, our outcomes did not include specialist health care, such as psychologist or psychiatrist treatment and associated hospitalizations, and treatment offered by private psychologists, or data on counseling services provided on campus, which typically do not require GP referral. While this suggests that our results are driven by mild to moderate symptoms, we note that more severe conditions typically will require at least one primary care consultation for referral to specialist care. To compensate for that our main measure leans towards lighter symptoms and conditions, we also measure effects on dispensed prescription drugs. However, the observation window for this outcome did not cover the period where we observed increases in mental health consultations. Further research is required to assess whether increases in dispensed anxiolytics, antidepressants, or sedatives followed suit when, or after, mental health consultations increased. Finally, the education database used to define our student population only includes information on enrollment and completed education, and we were therefore not able to exclude or censor students who dropped out during the period of study, an outcome that is associated with poor mental health [39]. To the extent that the pandemic increased dropout rates, and this had a negative effect of mental health in and of itself, dropout can be considered as a mechanism for our results, rather than a confounder that should be controlled for.

## Conclusion

In the current study comparing 12,501 pandemic students to 25,990 pre-pandemic students, we have shown a time-restricted increase in primary care consultations for anxiety and depression among students during the COVID-19 pandemic, and that the increase coincided with longer periods of strict infection control measures.

Concerns about the mental health of university students were frequently raised also prior to the pandemic. Our results may indicate that support systems for students should receive even more attention and may need to be upscaled due to the additional stress of the pandemic. Furthermore, it highlights that the benefits of social distancing measures should be weighed against the cost of potentially worsening mental health in vulnerable groups in future pandemic preparedness.

## Supplementary Information

**Additional file 1.**

## Data Availability

The data that support the findings of this study are available from Norwegian Directorate of Public health, Statistics of Norway, Norwegian Institute of Public Health and Norwegian Tax Administration but restriction apply to the availability of these data, which were used under license for the current study, and so are nor publicly available. Data are however available from the authors upon reasonable request and with permission of Norwegian Directorate of Public health, Statistics of Norway, Norwegian Institute of Public Health and Norwegian Tax Administration.

## References

[1] Insights for Education. Covid-19 and schools: What we can learn from six months of closures and reopening. 2022. Technical report, Insights For Education.

[2] The Norwegian Government. Tidslinje: myndighetenes håndtering av koronasituasjonen, https://www.regjeringen.no/no/tema/Koronasituasjonen/tidslinje-koronaviruset/id2692402/ (accessed 5.July 2022).

[3] Varma P, Junge M, Meaklim H (2021). Younger people are more vulnerable to stress, anxiety and depression during COVID-19 pandemic: a global cross-sectional survey. Prog Neuropsychopharmacol Biol Psychiatry.

[4] Son C, Hegde S, Smith A (2020). Effects of COVID-19 on college students’ mental health in the United States: interview survey study. J Med Internet Res.

[5] Arsandaux J, Montagni I, Macalli M (2021). Mental health condition of college students compared to non-students during COVID-19 lockdown: the CONFINS study. BMJ Open.

[6] Rezapour M, Dehzangi A, Saadati F (2022). Students’ negative emotions and their rational and irrational behaviors during COVID-19 outbreak. PLoS One.

[7] Li Y, Wang A, Wu Y (2021). Impact of the COVID-19 pandemic on the mental health of college students: a systematic review and meta-analysis. Front Psychol.

[8] Elmer T, Mepham K, Stadtfeld C (2020). Students under lockdown: Comparisons of students’ social networks and mental health before and during the COVID-19 crisis in Switzerland. PLoS One.

[9] Wang X, Hegde S, Son C (2020). Investigating mental health of US college students during the COVID-19 pandemic: cross-sectional survey study. J Med Internet Res.

[10] Naser AY, Dahmash EZ, Al-Rousan R (2020). Mental health status of the general population, healthcare professionals, and university students during 2019 coronavirus disease outbreak in Jordan: a cross-sectional study. Brain Behavior.

[11] Fretheim A, Helleve A, Løyland B (2021). Relationship between teaching modality and COVID-19, well-being, and teaching satisfaction (campus & corona): a cohort study among students in higher education. Public Health in Practice.

[12] Ma Z, Wang D, Zhao J (2022). Longitudinal associations between multiple mental health problems and suicidal ideation among university students during the COVID-19 pandemic. J Affect Disord.

[13] Šljivo A, Kačamaković M, Quraishi I (2020). Fear and depression among residents of Bosnia and Herzegovina during COVID-19 outbreak-internet survey. Psychiatr Danub.

[14] Hafstad GS, Sætren SS, Wentzel-Larsen T (2021). Adolescents’ symptoms of anxiety and depression before and during the Covid-19 outbreak–A prospective population-based study of teenagers in Norway. Lancet Regional Health-Europe.

[15] Koenig J, Kohls E, Moessner M, et al. The impact of COVID-19 related lockdown measures on self-reported psychopathology and health-related quality of life in German adolescents. Eur Child Adolesc Psychiatry. 2021:1–10. 10.1007/s00787-021-01843-1. 10.1007/s00787-021-01843-1 PMC8272610 34247297

[16] Li SH, Beames JR, Newby JM (2022). The impact of COVID-19 on the lives and mental health of Australian adolescents. Eur Child Adolesc Psychiatry.

[17] Thorisdottir IE, Asgeirsdottir BB, Kristjansson AL (2021). Depressive symptoms, mental wellbeing, and substance use among adolescents before and during the COVID-19 pandemic in Iceland: a longitudinal, population-based study. Lancet Psychiatry.

[18] Knapstad M, Sivertsen B, Knudsen AK (2021). Trends in self-reported psychological distress among college and university students from 2010 to 2018. Psychol Med.

[19] Kessler RC, Berglund P, Demler O (2005). Lifetime prevalence and age-of-onset distributions of DSM-IV disorders in the National Comorbidity Survey Replication. Arch Gen Psychiatry.

[20] Lewinsohn PM, Solomon A, Seeley JR (2000). Clinical implications of" subthreshold" depressive symptoms. J Abnorm Psychol.

[21] Fergusson DM, Woodward LJ (2002). Mental health, educational, and social role outcomes of adolescents with depression. Arch Gen Psychiatry.

[22] Nordmo M, Kinge JM, Reme B-A (2022). The educational burden of disease: a cohort study. Lancet Public Health.

[23] Jacobi F, Höfler M, Strehle J (2015). Twelve-months prevalence of mental disorders in the German Health Interview and Examination Survey for Adults-Mental Health Module (DEGS1-MH): a methodological addendum and correction. Int J Methods Psychiatr Res.

[24] Plana-Ripoll O, Musliner KL, Dalsgaard S (2020). Nature and prevalence of combinations of mental disorders and their association with excess mortality in a population-based cohort study. World Psychiatry.

[25] Meherali S, Punjani N, Louie-Poon S (2021). Mental health of children and adolescents amidst COVID-19 and past pandemics: a rapid systematic review. Int J Environ Res Public Health.

[26] Knox L, Karantzas GC, Romano D, et al. One Year On: What We Have Learned about the Psychological Effects of COVID-19 Social Restrictions–A Meta-Analysis. Current Opinion Psychol. 2022;46:101315. 10.1016/j.copsyc.2022.101315 PMC8907153 35398753

[27] Evensen M, Hart RK, Godøy AA, Hauge LJ, Lund IO, Knudsen AK, et al. Impact of the COVID-19 pandemic on mental healthcare consultations among children and adolescents in Norway: a nationwide registry study. Eur Child Adolesc Psychiatry. 2022;27:1–1. Advance online publication. 10.1007/s00787-022-02046-y.

[28] Hvide HK, Johnsen J (2022). COVID-19 and mental health: a longitudinal population study from Norway. Eur J Epidemiol.

[29] Helgeland J, Telle KE, Grøsland M (2021). Admissions to Norwegian hospitals during the COVID-19 pandemic. Scandinavian journal of public health.

[30] Knudsen AKS, Stene-Larsen K, Gustavson K (2021). Prevalence of mental disorders, suicidal ideation and suicides in the general population before and during the COVID-19 pandemic in Norway: a population-based repeated cross-sectional analysis. The Lancet Regional Health-Europe.

[31] Juul FE, Jodal HC, Barua I (2022). Mortality in Norway and Sweden during the COVID-19 pandemic. Scandinavian Journal of Public Health.

[32] Moradian S, Bäuerle A, Schweda A (2021). Differences and similarities between the impact of the first and the second COVID-19-lockdown on mental health and safety behaviour in Germany. J Public Health.

[33] Wang PS, Berglund PA, Olfson M (2004). Delays in initial treatment contact after first onset of a mental disorder. Health Serv Res.

[34] Krakowczyk JB, Planert J, Skoda E-M (2022). Pandemic fatigue, psychopathological risk factors, and vaccination attitudes during the COVID-19 pandemic in 2021–a network analysis. J Affect Disord Rep.

[35] Grøtan K, Sund ER, Bjerkeset O (2019). Mental health, academic self-efficacy and study progress among college students–The SHoT study, Norway. Front Psychol.

[36] Torvik FA, Ystrom E, Gustavson K (2018). Diagnostic and genetic overlap of three common mental disorders in structured interviews and health registries. Acta Psychiatr Scand.

[37] Alonso J, Liu Z, Evans-Lacko S (2018). Treatment gap for anxiety disorders is global: results of the world mental health surveys in 21 countries. Depress Anxiety.

[38] Thornicroft G, Chatterji S, Evans-Lacko S (2017). Undertreatment of people with major depressive disorder in 21 countries. Br J Psychiatry.

[39] Hjorth CF, Bilgrav L, Frandsen LS (2016). Mental health and school dropout across educational levels and genders: a 4.8-year follow-up study. BMC Public Health.

