# Supplementary material for: The COVID-19 pandemic’s impact on mental health care use among Norwegian students in higher education: a nation-wide register-based pre-post cohort study
Source: BMC Health Serv Res. 2022 Dec 10;22:1506. doi: 10.1186/s12913-022-08816-3 (PMC9736720; doi:10.1186/s12913-022-08816-3)
Supplement: Supplementary file 1 — Additional file 1. [file 12913_2022_8816_MOESM1_ESM.docx]

**Appendix to**

**Grøsland, Larsen, Reneflot, Hart (2022): The COVID-19 pandemic’s impact on the mental health of Norwegian students in higher education: a nation-wide register-based event study**

**Table A1. Descriptive statistics on group characteristics for pre-pandemic and pandemic students for the sample used for the prescribed drugs outcomes**

|  | **Pandemic cohort** | | **Pre-pandemic cohort** | |  |
| --- | --- | --- | --- | --- | --- |
| **Sample characteristics** |  | |  | |  |
| Persons, N | 12 501 | | 12 750 | |  |
| Age, mean (SD) | 19 (0) | | 19 (0) | |  |
| **Sex, N (%)** |  | |  | |  |
| Females | 7 878(63) | | 7 997 (62.7) | |  |
| Males | 4 623(37) | | 4 753 (37.2) | |  |
| **Birth country, N (%)** |  | |  | |  |
| Norway | 11 192(89.5) | | 11 541(90.5) | |  |
| Abroad | 1 309(10.5) | | 1 209(9.5) | |  |
|  |  | |  | |  |
| **Mental health outcomes, monthly %** | **Oct 2019-**  **Feb 2020** | **Mar 2020-**  **Aug 2021** | **Oct 2019-**  **Feb 2020^a^** | **Mar 2020-**  **Aug 2021^b^** |  |
| **Dispensed prescription drugs** |  |  |  |  | **ATC codes (prefix)** |
| Anxiolytics, antidepressants, sedatives | 0.8 | 0.94 | 0.83 | 1.01 | N05B*, N06A*, N05C* |
| Anxiolytics, antidepressants | 0.51 | 0.65 | 0.49 | 0.68 | N05B*, N06A* |
| Sedatives | 0.33 | 0.32 | 0.37 | 0.38 | N05C* |

**Table 1 notes:** Mental health outcomes show the monthly percentage of pandemic and pre-pandemic students with dispensed prescription drug (anxiolytic, antidepressant, or sedative). ^a,b^ October 2019-August 2021 refers to the measurement time (calendar month) for the pandemic students, i.e., measurements for the pre-pandemic students were made 12. ^a^October 2019-February 2020 corresponds to relative months -5 to -1 (pre-treatment period). ^b^ Mar 2020-Feb 2021 corresponds to relative months 0 to 11 (post-treatment period).

**Figure A1. The impact of the pandemic on mental health care use among students, absolute coefficients**


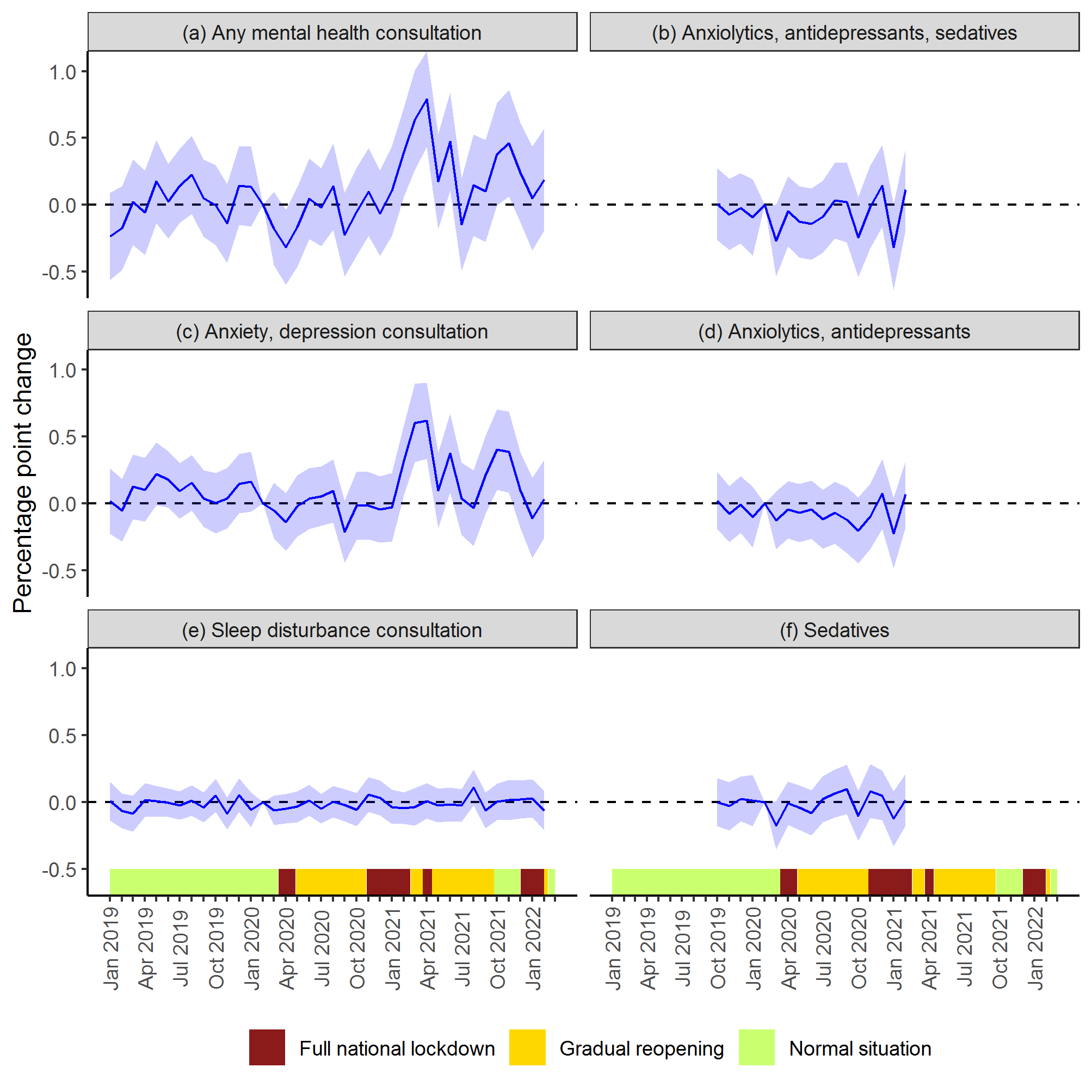


**Figure A1 note:** Results from event study models estimated separately for each outcome. Solid lines show monthly coefficients with corresponding 95 % confidence interval (shaded). Coefficients refer to the absolute change (measured in percentage points) in mental health care use among pandemic students, relative to pre-pandemic students. For more information on model specification see table note in table A2. The timeline of infection control measures, categorized by three levels of stringency, is illustrated on the x-axis (analogous to Figure 1).

| **Month** | **Any mental health consultation** | | **Anxiety/depression consultation** | | **Sleep disturbance consultation** | | **Anxiolytics, antidepressants, sedatives** | | **Anxiolytics/**  **antidepressants** | | **Sedatives** | |
| --- | --- | --- | --- | --- | --- | --- | --- | --- | --- | --- | --- | --- |
|  | **β (SE)** | **Rel. %** | **β (SE)** | **Rel. %** | **β (SE)** | **Rel. %** | **β (SE)** | **Rel. %** | **β (SE)** | **Rel. %** | **β (SE)** | **Rel. %** |
| 01.2019 | -0.23 (0.17) | -**22** | 0.02 (0.12) | 3 | 0.01 (0.07) | 4 |  |  |  |  |  |  |
| 02.2019 | -0.17 (0.16) | -17 | -0.05 (0.12) | -9 | -0.07 (0.07) | -46 |  |  |  |  |  |  |
| 03.2019 | 0.02 (0.16) | 2 | 0.12 (0.12) | 21 | -0.09 (0.07) | -59 |  |  |  |  |  |  |
| 04.2019 | -0.06 (0.16) | -5 | 0.1 (0.12) | 17 | 0.01 (0.06) | 10 |  |  |  |  |  |  |
| 05.2019 | 0.17 (0.16) | 16 | 0.22* (0.12) | 37 | 0.01 (0.06) | 5 |  |  |  |  |  |  |
| 06.2019 | 0.03 (0.14) | 2 | 0.18* (0.11) | 30 | -0.01 (0.05) | -4 |  |  |  |  |  |  |
| 07.2019 | 0.14 (0.14) | 13 | 0.09 (0.11) | 16 | -0.03 (0.05) | -17 |  |  |  |  |  |  |
| 08.2019 | 0.23 (0.15) | 21 | 0.15 (0.11) | 26 | 0.01 (0.06) | 8 |  |  |  |  |  |  |
| 09.2019 | 0.05 (0.15) | 5 | 0.04 (0.11) | 6 | -0.04 (0.06) | -27 |  |  |  |  |  |  |
| 10.2019 | 0 (0.15) | -0 | 0 (0.12) | 0 | 0.05 (0.06) | 34 | 0 (0.14) | 1 | 0.02 (0.11) | 4 | 0 (0.09) | 0 |
| 11.2019 | -0.14 (0.15) | -13 | 0.04 (0.11) | 7 | -0.08 (0.06) | -58 | -0.07 (0.14) | -9 | -0.08 (0.11) | -16 | -0.03 (0.09) | -9 |
| 12.2019 | 0.15 (0.15) | 13 | 0.15 (0.11) | 25 | 0.05 (0.06) | 36 | -0.02 (0.13) | -3 | -0.01 (0.11) | -12 | 0.02 (0.09) | 7 |
| 01.2020 | 0.14 (0.15) | 13 | 0.16 (0.11) | 27 | -0.06 (0.07) | -41 | -0.09 (0.15) | -12 | -0.1 (0.12) | -20 | 0.01 (0.1) | 3 |
| 02.2020 | 0 | 0 | 0 | 0 | 0 | 0 | 0 | 0 | 0 | 0 | 0 | 0 |
| 03.2020 | -0.18 (0.14) | -16 | -0.05 (0.11) | -9 | -0.06 (0.06) | -42 | -0.27* (0.14) | -34 | -0.13 (0.11) | -25 | -0.17* (0.09) | -53 |
| 04.2020 | -0.32** (0.14) | -29 | -0.14 (0.11) | -23 | -0.05 (0.06) | -35 | -0.05 (0.13) | -6 | -0.05 (0.11) | -9 | -0.01 (0.08) | -3 |
| 05.2020 | -0.17 (0.15) | -15 | -0.02 (0.12) | -3 | -0.03 (0.06) | -24 | -0.13 (0.14) | -16 | -0.07 (0.11) | -14 | -0.04 (0.08) | -12 |
| 06.2020 | 0.05 (0.15) | 4 | 0.04 (0.12) | 6 | 0.01 (0.06) | 8 | -0.14 (0.14) | -18 | -0.05 (0.11) | -9 | -0.08 (0.09) | -24 |
| 07.2020 | -0.02 (0.15) | -2 | 0.05 (0.11) | 9 | -0.05 (0.06) | -33 | -0.09 (0.14) | -11 | -0.12 (0.11) | -23 | 0.02 (0.09) | 7 |
| 08.2020 | 0.14 (0.16) | 13 | 0.09 (0.12) | 16 | 0 (0.06) | 3 | 0.03 (0.14) | 4 | -0.07 (0.12) | -14 | 0.06 (0.09) | 19 |
| 09.2020 | -0.22 (0.16) | -21 | -0.21* (0.12) | -36 | -0.02 (0.06) | -16 | 0.02 (0.15) | 2 | -0.12 (0.13) | -24 | 0.1 (0.09) | 29 |
| 10.2020 | -0.05 (0.17) | -5 | -0.02 (0.13) | -3 | -0.06 (0.06) | -39 | -0.24 (0.15) | -30 | -0.2 (0.13) | -40 | -0.1 (0.1) | -31 |
| 11.2020 | 0.1 (0.17) | 9 | -0.02 (0.13) | -3 | 0.06 (0.07) | 38 | -0.02 (0.16) | -2 | -0.1 (0.12) | -20 | 0.08 (0.1) | 25 |
| 12.2020 | -0.06 (0.16) | -6 | -0.04 (0.13) | -8 | 0.03 (0.07) | 22 | 0.14 (0.16) | 18 | 0.07 (0.13) | 14 | 0.05 (0.09) | 15 |
| 01.2021 | 0.11 (0.17) | 10 | -0.03 (0.13) | -5 | -0.04 (0.07) | -25 | -0.32* (0.16) | -40 | -0.22* (0.13) | -44 | -0.12 (0.1) | -38 |
| 02.2021 | 0.4** (0.17) | 37 | 0.32** (0.13) | 54 | -0.05 (0.06) | -32 | 0.12 (0.16) | 15 | 0.07 (0.13) | 13 | 0.02 (0.1) | 5 |
| 03.2021 | 0.63*** (0.19) | 59 | 0.6*** (0.15) | 102 | -0.04 (0.07) | -25 |  |  |  |  |  |  |
| 04.2021 | 0.79*** (0.18) | 73 | 0.62*** (0.14) | 104 | 0.01 (0.07) | 5 |  |  |  |  |  |  |
| 05.2021 | 0.17 (0.18) | 16 | 0.1 (0.14) | 16 | -0.02 (0.06) | -17 |  |  |  |  |  |  |
| 06.2021 | 0.47** (0.19) | 44 | 0.38** (0.15) | 63 | -0.02 (0.06) | -11 |  |  |  |  |  |  |
| 07.2021 | -0.15 (0.18) | -14 | 0.04 (0.14) | 6 | -0.02 (0.06) | -17 |  |  |  |  |  |  |
| 08.2021 | 0.15 (0.19) | 14 | -0.03 (0.14) | -6 | 0.11 (0.07) | 76 |  |  |  |  |  |  |
| 09.2021 | 0.1 (0.19) | 10 | 0.21 (0.15) | 36 | -0.06 (0.07) | -42 |  |  |  |  |  |  |
| 10.2021 | 0.38* (0.2) | 35 | 0.4*** (0.15) | 67 | 0 (0.07) | 2 |  |  |  |  |  |  |
| 11.2021 | 0.46** (0.2) | 43 | 0.38** (0.15) | 65 | 0.02 (0.08) | 10 |  |  |  |  |  |  |
| 12.2021 | 0.24 (0.19) | 22 | 0.1 (0.14) | 17 | 0.02 (0.07) | 13 |  |  |  |  |  |  |
| 01.2022 | 0.05 (0.2) | 4 | -0.11 (0.15) | -18 | 0.03 (0.07) | 19 |  |  |  |  |  |  |
| 02.2022 | 0.19 (0.19) | 18 | 0.03 (0.15) | 6 | -0.06 (0.08) | -45 |  |  |  |  |  |  |

**Table A2. Event study estimates of the monthly difference in mental health consultations and dispensed prescribed drugs.**

**Table A2 notes:** Results from event study models (β) quantify the change in mental health outcomes (measured as change in percentage points), controlling for age, year of enrollment and a dummy variable for the duration variable in month. March 2020 (t-1) is set to reference period. Standard errors (SEs) are clustered on individuals. In addition to the presentation of results in absolute terms, relative differences in percent (Rel. %) are also presented, calculated by dividing the absolute estimate (and corresponding standard error) for each of the post-periods by the monthly average health outcome for the pandemic students in the period prior to the pandemic (January 2019-February 2020 for consultations and October 2019-February 2020 for dispensed prescription drugs). Stars indicate confidence levels (*p≤0.1; **p≤0.05; ***p≤0.01).
